# Immune checkpoint inhibitor-related kelch-like protein 11-IgG cerebellitis successfully treated with efgartigimod as rescue therapy: a case report

**DOI:** 10.3389/fimmu.2026.1864182

**Published:** 2026-06-03

**Authors:** Yi-Xiao Li, Zhong-Mian Ma

**Affiliations:** 1 Department of Neurology, Ningbo Taikang Hospital, Ningbo, China; 2 Department of Rehabilitation Medicine, Ningbo Taikang Hospital, Ningbo, China

**Keywords:** cerebellitis, efgartigimod, immune checkpoint inhibitor, immunotherapy, kelch-like protein-11

## Abstract

**Background:**

While immune checkpoint inhibitors (ICIs) have revolutionized cancer therapy, neurological immune-related adverse events (n-irAEs) associated with these agents are increasingly recognized. However, kelch-like protein-11(KLHL11)-IgG cerebellitis has not been reported as an n-irAE.

**Case presentation:**

We report a case of a 53-year-old man with lung adenocarcinoma treated with tislelizumab who developed KLHL11-IgG cerebellitis. First-line immunotherapy, comprising intravenous methylprednisolone and immunoglobulin, failed to improved his symptoms. Subsequent administration of efgartigimod (10 mg/kg weekly for 4 doses) led to seroconversion of KLHL11-IgG and marked clinical recovery.

**Conclusion:**

This is the first report of efgartigimod as a successful rescue therapy for isolated ICI-related KLHL11-IgG cerebellitis. These findings support efgartigimod as a promising treatment option for this condition.

## Introduction

The advent of immune checkpoint inhibitors (ICIs) has revolutionized cancer therapy over the past decade. As the clinical use of ICIs expands, neurological immune-related adverse events (n-irAEs) associated with these treatments are increasingly recognized.

Previous studies have reported that n-irAEs affect approximately 1% of patients receiving ICI monotherapy and 2%–3% of those receiving ICI combination therapy (1, 2). Although less frequent than other ICI-related toxicities, n-irAEs can lead to significant disability or death, underscoring the need for their prompt recognition and management.

ICI-associated cerebellitis remains a rare but potentially disabling complication. Isolated kelch-like protein-11(KLHL11)-IgG cerebellitis has not been previously described as an n-irAE, and its optimal management remains unclear. Here, we report a case of ICI-related KLHL11-IgG cerebellitis in which efgartigimod was administered after the patient failed to respond to intravenous methylprednisolone (IVMP) and intravenous immunoglobulin (IVIG), leading to a favorable therapeutic outcome.

## Case presentation

A 53-year-old man was newly diagnosed with stage IV (T3N2M1a) lung adenocarcinoma. His past medical history was notable for chronic active smoking, a history of pulmonary tuberculosis, and coronary heart disease. He had undergone coronary artery stenting in 2018 with good postprocedural recovery. Pathological examination of the lung tumor showed thyroid transcription factor 1 positivity, with 70% of tumor cells testing positive for programmed death-ligand 1; anaplastic lymphoma kinase, c-ros oncogene 1, and epidermal growth factor receptor were all wild-type. He was initiated on combination therapy with tislelizumab (200 mg IV [intravenously] every three weeks), cisplatin (75 mg/m² IV every three weeks), and pemetrexed (500 mg/m² IV every three weeks). After three cycles, a chest computed tomography (CT) scan showed a partial response, with reduction in the size of the primary lung tumor.

Eleven days after the fourth cycle of combination therapy, he presented with fever, fatigue, headache, anorexia, and vomiting. Brain magnetic resonance imaging (MRI) showed no evidence of metastatic disease, and abdominal CT along with gastroenterological endoscopy revealed unremarkable findings. Subsequently, two weeks after the sixth cycle of tislelizumab, he developed slurred speech, dizziness, gait instability, and recurrent vomiting. His symptoms were initially attributed to acute stroke, although the original MRI reports were unavailable. He was treated with antiplatelet therapy consisting of enteric-coated aspirin and clopidogrel hydrogen sulphate tablet for eight days. However, progressive neurological deterioration prompted referral to our center.

On admission, neurological examination revealed dysarthria, ocular dysmetria, gaze-evoked nystagmus, appendicular ataxia, titubations, and wide-based gait. He was unable to stand without assistance. The maximal walking distance with crutches was 100 m, with multiple recent falls. Cognitive, sensory and motor functions were all normal. The SARA (Scale for the Assessment and Rating of Ataxia) score was 15. The modified Rankin scale (mRS) score was 4. Routine blood analysis showed no clinically relevant abnormalities. All systemic autoantibodies were all negative (**Supplementary Table 1**). Cerebrospinal fluid (CSF) analysis revealed an elevated white blood cell count (7.0 cells/µL; 80% lymphocytes), elevated protein level (59.0 mg/dL), a normal IgG index (0.66), and no CSF-restricted oligoclonal bands. Lumbar puncture was repeated three times to rule out infectious or neoplastic causes; no evidence of infection (bacterial, viral, or fungal) or cancer dissemination was found, and bacterial cultures of CSF were negative. Cell-based assay (CBA) for demyelination-associated antibodies and autoimmune encephalitis-related antibodies was negative in both serum and CSF. CBA for onconeural antibodies demonstrated KLHL11-IgG positivity in serum (titer 1:100; reference range: >1:10) and CSF (titer 1:1; reference range: >1:1) (details are provided in **Supplementary Table 1**; **Supplementary Figure 1**).

Two days after admission to our center, the patient underwent brain MRI, which revealed T2/fluid-attenuated inversion recovery hyperintensity in the right cerebellar hemisphere, with patchy enhancement in the corresponding regions (**Figure 1**). Positron emission tomography-CT revealed no tumor metastasis. Scrotal ultrasound showed small bilateral epididymal cysts but no testicular mass.

**Figure 1:**
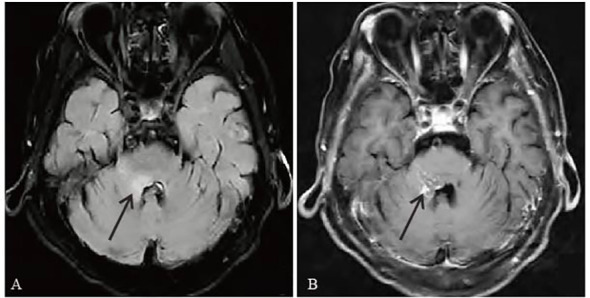
MRI findings of a patient with ICI-related KLHL11-IgG cerebellitis. **(A)** Axial MRI revealed T2/FLAIR hyperintensity in the right cerebellar hemisphere. **(B)** Axial MRI revealed patchy enhancement in the right cerebellar hemisphere. FLAIR, fluid-attenuated inversion imaging; ICI, immune checkpoint inhibitor; IgG, immunoglobulin; KLHL11, Kelch-like protein-11; MRI, magnetic resonance imaging.

Based on these findings, the patient was diagnosed with ICI-related KLHL11-IgG cerebellitis. After a multidisciplinary team consultation, tislelizumab was immediately discontinued, while cisplatin and pemetrexed were continued to maintain anti-tumor efficacy. IVMP (1,000 mg/day for five days, tapered to 500 mg/day for five days, then 250 mg/day for three days, and subsequently 120 mg/day for three days) was initiated concurrently with IVIG (0.4 g/kg/day for five days).

Following IVMP and IVIG, his dizziness and vomiting improved slightly, but severe gait ataxia and disequilibrium persisted, leaving him unable to walk independently (SARA 14; mRS 4). Plasma exchange (PLEX) was considered but could not be performed due to limited blood product availability at our institution. After obtaining informed consent, the patient received efgartigimod (10 mg/kg IV weekly for four weeks) as add-on rescue therapy.

Cerebellar symptoms began to improve after the second efgartigimod infusion. By day 5 post-infusion, vomiting and dizziness had markedly decreased, and gait ataxia had substantially improved. His maximal walking distance with crutches was 450 m, without falls (SARA 8; mRS 3). On day 6 after the third infusion, he could walk 700 m without assistance, with residual unsteadiness (SARA 7; mRS 3). Two weeks after the fourth infusion, he walked independently with a SARA score of 5 and an mRS score of 2, and was subsequently discharged.

At the six-month follow-up, the patient reported occasional dizziness and mild slurred speech (SARA 2; mRS 1) and remained on low-dose prednisone (5 mg daily) maintenance without relapse. At the one-year follow-up, he had only mild dysarthria (SARA 1; mRS 1) (**Figure 2**). Serum KLHL11-IgG testing was negative by CBA, and brain MRI confirmed complete resolution of the cerebellar inflammatory lesions. His lung adenocarcinoma remains in remission on cisplatin and pemetrexed. The patient remains under regular surveillance. The clinical findings and disease course are summarized in **Figure 2**.

**Figure 2:**
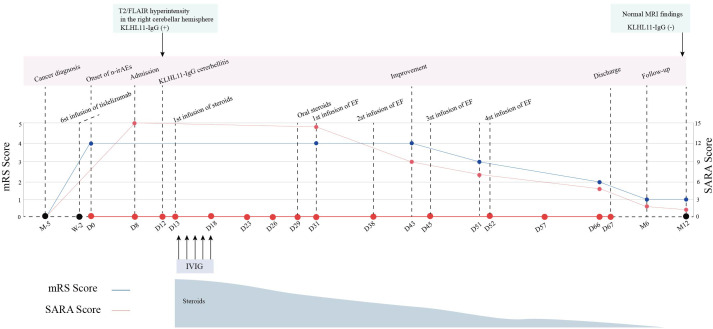
Clinical findings and course of disease. D, day; EF, efgartigimod; FLAIR, fluid-attenuated inversion imaging; IgG, immunoglobulin; IVIG, intravenous immunoglobulin; KLHL11, Kelch-like protein-11; MRI, magnetic resonance imaging; mRS, modified Rankin scale; M, month; n-irAE, immune checkpoint inhibitor-related adverse event; SARA, Scale for the Assessment and Rating of Ataxia; W, week.

## Discussion

Cerebellitis is a known complication of ICI therapy. A recent systematic review included 46 cases of ICI-associated cerebellar involvement, 64% of which were associated with anti-PD-1 therapy (3). Autoantibodies were detected in 61.9% of patients, most commonly anti-neuronal intermediate filament, anti-Hu, anti-GAD65, and anti-CRMP5 antibodies (3). Although KLHL11-IgG rhombencephalitis has been reported twice as an n-irAE, occurring with nivolumab for esophageal cancer and avelumab for Merkel cell carcinoma, respectively (4, 5), isolated KLHL11-IgG cerebellitis has not been reported as an n-irAE. Herein, we report a patient with lung adenocarcinoma who developed isolated KLHL11-IgG cerebellitis potentially associated with ICI exposure, and finally achieved marked clinical improvement following efgartigimod rescue therapy.

KLHL11 antibodies were first identified in 2019 as a biomarker of paraneoplastic neurological syndrome (PNS), predominantly associated with testicular germ cell tumors (78.7%), followed by ovarian or testicular teratoma (14.8%), small cell lung cancer, and lung adenocarcinoma (6–8). Most patients had a cerebellar syndrome with or without rhombencephalitis, with the most frequent symptoms being ataxia (73.2%), vertigo (41.9%), oculomotor disturbances (31.3%), and hearing disorders (27.7%) (9). In the 2021 updated diagnostic criteria for PNS, KLHL11 antibody was classified as a high-risk antibody, and rapidly progressive cerebellar syndrome (including cerebellitis) as a high-risk neurological phenotype with >70% cancer association (10). Although KLHL11 immunoreactivity in lung tumor tissues was not assessed by immunohistochemistry, our patient met the criteria for probable PNS (PNS-Care Score = 7) based on clinical, serological, and oncological findings.

Notably, the patients was diagnosed with lung adenocarcinoma without definitive testicular space-occupying lesions, which deviates from the classic association between KLHL11-IgG and testicular germ cell tumors. This raises a critical question regarding the conceptual boundary between ICI-related PNS and classical (spontaneous) PNS. It remains challenging to definitively distinguish whether this case represents a *de novo* n-irAE directly induced by tislelizumab, or a pre-existing subclinical PNS that was unmasked or accelerated by ICI-induced immune activation. We hypothesized that the tumor itself may have triggered subclinical production of KLHL11-IgG, but this process per se was not sufficient to elicit overt neurological symptoms. Subsequent ICI administration disrupted immune self-tolerance, enhanced antitumor immune responses, and promoted the release and presentation neuronal antigens, thereby precipitating the rapid onset and progression of symptomatic KLHL11-IgG cerebellitis. Patients with pre-existing autoantibodies may increase the risk of n-irAEs (11). Prospective studies are warranted to monitor the presence and titers of onconeural antibodies before, during, and after ICI treatment, and to correlate these dynamics with the development of neurological syndromes.

Diagnostic delayed could result in inappropriate continuation of ICI treatment and late introduction of immunotherapy. The MATCH score (1 point for male, 1 for ataxia or other cerebellar symptoms, 2 for testicular cancer, 1 for cancer of other types, 1 for hearing alterations) was proposed to guide KLHL11 antibody testing, with a cutoff of ≥4 (12). A previous study reported 78% sensitivity and 99% specificity for this score (9). Our patient scored 3 (male, ataxia, and other cancer), below the cutoff, suggesting that exclusive reliance on the MATCH score may miss cases of ICI-associated KLHL11-IgG cerebellitis. We therefore suggest considering KLHL11 antibody testing in ICI-treated patients presenting with acute or subacute ataxia of unclear etiology.

Management of KLHL11-IgG rhombencephalitis/cerebellitis is challenging due to its treatment-refractory nature. IVMP followed by oral steroids and IVIG represents the most commonly used first-line immunotherapy, while cyclophosphamide (CTX) is the most frequently administered second-line agent (6, 9). A recent systematic review including 79 KLHL11-IgG associated PNS patients treated showed 43% experienced further neurological deterioration, 36.7% remained stable, and only 20% achieved clinical improvement (9). The therapeutic strategy for ICI-related KLHL11-IgG rhombencephalitis/cerebellitis remains poorly standardized, and current management is primarily guided by severity-based grading systems recommended by the American Society of Clinical Oncology (ASCO) guidelines (13). Specifically, Grade 1 (asymptomatic) warrants close monitoring without ICI interruption; Grade 2 (moderate symptoms) warrants ICI discontinuation plus high-dose corticosteroids; and Grades 3–4 (severe symptoms, without or with life-threatening risks) warrant high-dose corticosteroids combined with IVIG or plasma exchange (13). The first documented case of ICI-related KLHL11-IgG rhombencephalitis was treated with CTX and remained clinically stable (4). In contrast, another patient diagnosed with ICI-related KLHL11-IgG rhombencephalitis showed poor response to multiple immunosuppressive agents consisting of IVMP, IVIG, PLEX, and CTX, and finally died (5). In the present case, first-line treatment consisting of IVMP and IVIG failed to achieve obvious clinical improvement. These findings highlight the refractory nature of ICI-related KLHL11-IgG rhombencephalitis/cerebellitis, and emphasize the need for more effective therapeutic approaches. PLEX was unavailable at our center; consequently, efgartigimod was selected as a novel rescue therapy. Efgartigimod, a neonatal Fc receptor (FcRn) inhibitor, reduces circulating IgG levels, suppresses FcRn-mediated immune complex presentation, and decreases proinflammatory cytokine release (14). It has shown promising efficacy in refractory paraneoplastic encephalitis and induced complete remission in a case of KLHL11-IgG encephalitis (7, 15). These observations suggest that efgartigimod, when used as an add-on therapy to steroids, can induce rapid and significant clinical improvement with a favorable safety profile. However, specific regimens (including dosage per administration and treatment duration) require further investigation. Our patient achieved favorable neurological recovery following efgartigimod, suggesting its potential efficacy for KLHL11-IgG encephalitis/cerebellitis, including ICI-related cases.

## Conclusion

We report a case of ICI-related KLHL11-IgG cerebellitis successfully treated with the FcRn inhibitor efgartigimod after failure of conventional immunotherapy. This observation highlights the potential of FcRn inhibitors in this refractory condition and underscores the importance of early recognition. Larger cohort studies are needed to confirm efficacy.

## Data Availability

The original contributions presented in the study are included in the article/**Supplementary Material**. Further inquiries can be directed to the corresponding author.

## Supplementary material

Supplementary Table 1 Hematological and cerebrospinal fluid tests for diagnostic evaluation.

Supplementary Figure 1 The patient’s **(A)** serum (titer: 1:100) and **(B)** cerebrospinal fluid (titer: 1:1) were positive for anti-KLHL11-IgG by cell-based assay.
