# Supplementary material for: Immune checkpoint inhibitor-related kelch-like protein 11-IgG cerebellitis successfully treated with efgartigimod as rescue therapy: a case report
Source: Front Immunol. 2026 Jun 3;17:1864182. doi: 10.3389/fimmu.2026.1864182 (PMC13272319; doi:10.3389/fimmu.2026.1864182)
Supplement: Supplementary Table 1 — Hematological and cerebrospinal fluid tests for diagnostic evaluation. [file Table1.docx]

**Supplementary Table 1 Hematological and CSF tests for diagnostic evaluation**

| **General exam** | **Blood and serum** | **CSF** |
| --- | --- | --- |
|  | Hemoglobin: 148 g/L | Proteins: 65 mg/dL |
|  | CRP: 30 mg/L | Glucose: 2.7 mmol/L |
|  | ESR: 22 mmol/L | LDH: 2.5 mmol/L |
|  | Proteins: 79 g/L | WBC: 7.0 cells/µL |
|  | Creatinine: 76 mmol | Lymphocytes: 80% |
|  | Glycemia: 6.3 mmol/L | Normal IgG index (0.64), absent |
|  | Calcium: 2.6 mmol/L | OCBs: negative |
|  | ALT: 35 UI/L |  |
|  | AST: 40 UI/L |  |
|  | OCBs: negative |  |
| Infection screening | Blood cultures: sterile | VZV, EBV, CMV, HSV 1-2, HHV-6, Cryptococcus, Streptococcus agalactiae, Streptococcus pneumoniae. Neisseria meningitis: negative by PCR |
| Autoimmune screening | Anti-ANA, anti-SSA, anti-SSB, anti-ANCA, RF, anti-CCP: negative |  |
|  | Autoimmune antibody panel (antibodies against IgLON5, DPPX, GlyR1, DRD2, mGluR5, NMDAR, AMPA1, AMPA2, LGI1, CASPR2, GABAA, GABAB, mGluR1, GAD65, and Neurexin-3α) : negative (by CBA) | Autoimmune antibody panel (antibodies against IgLON5, DPPX, GlyR1, DRD2, mGluR5, NMDAR, AMPA1, AMPA2, LGI1, CASPR2, GABAA, GABAB, mGluR1, GAD65, and Neurexin-3α) : negative (by CBA) |
|  | Onconeuronal antibody panel (antibodies against Hu, Ri, Yo, Ma1/2, SOX1, Recoverin, Amphiphysin, CRMP5/CV2): negative (by CBA) | Onconeuronal antibody panel (antibodies against Hu, Ri, Yo, Ma1/2, SOX1, Recoverin, Amphiphysin, CRMP5/CV2): negative (by CBA) |
|  | Anti-AQP4, anti-MOG, anti-GFAP: negative (by CBA) | Anti-AQP4, anti-MOG, anti-GFAP: negative (by CBA) |
|  | Anti-KLHL11: positive (by CBA) | Anti-KLHL11: positive (by CBA) |

**Abbreviations:** ALT, alanine aminotransferase; ANA, anti-nuclear antibody; ANCA, anti-neutrophil cytoplasmic antibody; AST, aspartate aminotransferase; CBA, cell-based assay; CCP, cyclic citrullinated peptide; CMV, cytomegalovirus; CRMP5, collapsin response mediator protein 5; CRP, C-reactive protein; CSF, cerebrospinal fluid; EBV, Epstein-Barr virus; ESR, erythrocyte sedimentation rate; GAD65, glutamic acid decarboxylase 65; HHV-6, human herpesvirus-6; HSV, herpes simplex virus; IgG, immunoglobulin G; KLHL11, Kelch-like protein 11; LDH, lactate dehydrogenase; mGluR1, metabotropic glutamate receptor 1; OCB, oligoclonal band; PCR, polymerase chain reaction; RF, rheumatoid factor; SSA, Sjögren syndrome antigen A; SSB, Sjögren syndrome antigen B; VZV, varicella-zoster virus; WBC, white blood cell.
