# Supplementary figures and images for: Immune checkpoint inhibitor-related kelch-like protein 11-IgG cerebellitis successfully treated with efgartigimod as rescue therapy: a case report
Source: Front Immunol. 2026 Jun 3;17:1864182. doi: 10.3389/fimmu.2026.1864182 (PMC13272319; doi:10.3389/fimmu.2026.1864182)

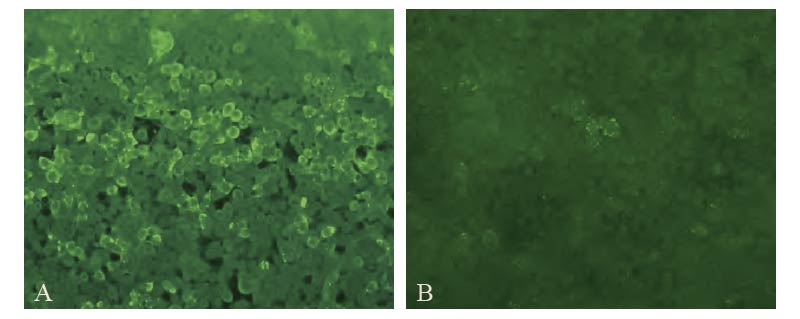

Supplement: Supplementary Figure 1 — The patient’s (A) serum (titer: 1:100) and (B) cerebrospinal fluid (titer: 1:1) were positive for anti-KLHL11-IgG by cell-based assay. [file Image1.jpeg]
